# Health Risk Assessment of Ortho-Toluidine Utilising Human Biomonitoring Data of Workers and the General Population

**DOI:** 10.3390/toxics10050217

**Published:** 2022-04-25

**Authors:** Pasi Huuskonen, Spyros Karakitsios, Bernice Scholten, Joost Westerhout, Dimosthenis A. Sarigiannis, Tiina Santonen

**Affiliations:** 1 Finnish Institute of Occupational Health, 00032 Työterveyslaitos, Finland; tiina.santonen@ttl.fi; 2 Environmental Engineering Laboratory, Department of Chemical Engineering, Aristotle University of Thessaloniki, 54124 Thessaloniki, Greece; spyrosk@auth.gr (S.K.); sarigiannis@auth.gr (D.A.S.); 3 HERACLES Research Center on the Exposome and Health, Center for Interdisciplinary Research and Innovation, 57001 Thessaloniki, Greece; 4 The Netherlands Organisation for Applied Scientific Research (TNO), 3508 TA Utrecht, The Netherlands; bernice.scholten@tno.nl (B.S.); joost.westerhout@tno.nl (J.W.)

**Keywords:** ortho-toluidine, biomonitoring, urinary mass-balance, PBPK modelling, occupational exposure, general population

## Abstract

The aim of this work was to demonstrate how human biomonitoring (HBM) data can be used to assess cancer risks for workers and the general population. Ortho-toluidine, OT (CAS 95-53-4) is an aniline derivative which is an animal and human carcinogen and may cause methemoglobinemia. OT is used as a curing agent in epoxy resins and as intermediate in producing herbicides, dyes, and rubber chemicals. A risk assessment was performed for OT by using existing HBM studies. The urinary mass-balance methodology and generic exposure reconstruction PBPK modelling were both used for the estimation of the external intake levels corresponding to observed urinary levels. The external exposures were subsequently compared to cancer risk levels obtained from the evaluation by the Scientific Committee on Occupational Exposure Limits (SCOEL). It was estimated that workers exposed to OT have a cancer risk of 60 to 90:10^6^ in the worst-case scenario (0.9 mg/L in urine). The exposure levels and cancer risk of OT in the general population were orders of magnitude lower when compared to workers. The difference between the output of urinary mass-balance method and the general PBPK model was approximately 30%. The external exposure levels calculated based on HBM data were below the binding occupational exposure level (0.5 mg/m^3^) set under the EU Carcinogens and Mutagens Directive.

## 1. Introduction

Aniline and many of its derivatives are known or suspected human carcinogens. Ortho-toluidine, OT (CAS 95-53-4), also known as 2-aminotoluene, is one of these aniline derivatives which is manufactured and/or imported in the European Economic Area in quantities of 10,000–100,000 tonnes per annum [1]. OT is mainly used as a curing agent in epoxy resins, an intermediate in producing azo dyes and pigments, acid-fast dyestuffs, triarylmethane dyes, sulphur dyes, indigo compounds, photographic dyes, synthetic rubber, and rubber vulcanising chemicals, and an intermediate in the manufacture of herbicides [1,2].

OT is an animal and human carcinogen, classified as a category Carcinogenic 1B according to harmonised classification and labelling in European Union, EU (Classification, Labelling and Packaging (CLP) regulation 1272/2008). Other CLP classifications of OT are Acute Toxicity 3 for oral and inhalation toxicity, Eye Irritation 2 for irritative properties and Aquatic Acute 1 for environmental toxicity. In addition, OT may cause methemoglobinemia in humans [1,3]. Several aniline derivatives can be found on the candidate list of substances of very high concern (SVHCs) and the list of substances restricted under Registration, Evaluation, Authorisation and Restriction of Chemicals (REACH) EU regulation. OT has been added to the candidate list for eventual inclusion in Annex XIV to REACH [4]. Inclusion in annex XIV to REACH means that future use of OT requires authorisation in the EU. For authorisation purposes, users of OT need to demonstrate the adequate control of exposure by providing reliable and representative exposure information from their uses. Human biomonitoring (HBM) data could be a useful data source if measured biomarker levels can be related to cancer risk assessment.

OT is rapidly absorbed via the gastrointestinal tract, and is rapidly distributed, metabolised, and excreted (mainly via urine) in rats. The saturation of metabolic pathways is not apparent in the urinary excretion data. Absorption via skin as well as via respiratory tract has been demonstrated by acute toxicity studies and in occupationally exposed humans [1,2]. Metabolism studies have showed that N-acetylation and hydroxylation of the aromatic ring of OT as well as sulphate and glucuronide conjugation are the major metabolic pathways in rats. The human metabolic pathways are expected to be similar to those reported in experimental animals based on studies of other aromatic amines and knowledge of the key metabolising enzymes [5].

Occupational exposure to OT can be measured either by measuring air concentrations or by biomonitoring. Since dermal uptake may contribute significantly to systemic exposure to OT, biomonitoring can give a better picture on the total exposure than air monitoring. Both urinary OT and OT haemoglobin adduct analyses have been used to monitor occupational exposure to OT. However, the measurement of urinary excretion of total (free and conjugated) OT after hydrolysis is currently the most used method [1]. Since OT is a genotoxic carcinogen, there is no health-based biological limit value. In the EU, a binding occupational exposure limit value (BOELV) of 0.1 ppm (0.5 mg/m^3^) has been set for OT [6]. This is not a purely health-based value but considers also socio-economic aspects.

Data on urinary OT levels of the general population have been used to set a biological guidance value (BGV) for urinary OT [1]. Based on the studies by Kütting et al. [7] and Weiss and Angerer [8], the 95th percentile of urinary OT (free and conjugated) among the non-smoking general population is approximately 0.2 μg/L which has been set as a biological guidance value (BGV) for workers in Germany for OT measured from post-shift urinary samples in the end of the working week [9]. Since smoking increases urinary OT levels, this value applies only for non-smokers or can be considered only after abstaining from smoking.

The aim of this work was to demonstrate how biomonitoring data can be used to assess cancer risks for workers and the general population. Using available information on the dose–response of OT carcinogenicity and published human biomonitoring (HBM) data, a risk assessment was conducted by applying a simple urinary mass-balance-based approach and a more refined exposure reconstruction algorithm coupled with a physiologically based pharmacokinetic (PBPK) model to convert urinary total OT levels as external daily intake.

## 2. Materials and Methods

The hazard and dose–response assessment of OT was based on the existing risk assessment from the Scientific Committee on Occupational Exposure Limits, SCOEL [1]. Since the SCOEL assessment covers only occupational exposure, cancer dose response for the general population was derived using a published benchmark dose lower limit (BMDL) level for OT as a starting point and using the general approach for cancer dose response setting described in REACH guidance R.8 [10]. A PubMed literature search was conducted for the past 20 years on OT in combination with occupation*, biomonitoring, and smoking, since OTs exist in cigarette smoke, to retrieve available occupational and general population biomonitoring data. Since the available HBM studies on the occupational exposure to OT were very limited in number, all relevant occupational HBM studies were included in the risk assessment. In the case of general population exposure assessment, only European HBM studies were included.

External exposure levels of OT were calculated from biomonitoring data of the general population and workers using a urinary mass-balance approach and by PBPK exposure reconstruction model to evaluate the cancer risk using the dose-response data published by SCOEL [1].

### 2.1. Urinary Mass-Balance Approach

The urinary mass-balance approach, as described by Angerer et al. [11], was used as a first-tier approach for the calculation of corresponding external intake levels from biomonitoring data. For this, the following formulas were used:

Css = (D × BW × FUE)/V24 or D = (Css × V24)/(FUE × BW)

Here, D is the external dose as mg/kg bw, Css is the urinary level of the substance at steady state (mg/L), V24 is the estimated average 24 h urinary volume (L), and FUE is the mass of OT, including hydrolysed conjugate metabolites, excreted in urine during 24 h per mass of parent compound ingested (percentage).

In the calculations, OT urinary levels (Css) were assumed to represent a steady-state level, and 75% of the dose was assumed to be excreted in urine as measured parent compound, including hydrolysed conjugate metabolites (FUE), which was based on urine excretions observed in s.c. dosed rats after 24 h [2,5]. A value of 1.5 L/day was used as the 24 h urinary volume (V24) and 70 kg as the average body weight (BW).

As an example, the estimated urinary OT level corresponding to the BOELV 0.1 ppm (0.5 mg/m^3^ = 0.07 mg/kg) assuming default 10 m^3^ inhalation volume during working day, [10] is 2.5 mg/L (0.07 × 70 × 0.75/1.5). Conversely, the urinary OT BGV of 0.2 μg/L can be estimated to 5.7 ng/kg of OT by using parameters presented above (0.2 × 1.5/0.75 × 70).

### 2.2. PBPK Modelling

A generic PBPK model implemented in the INTEGRA platform for integrative exposure and risk modelling [12,13] was used to reconstruct external exposures of OT from HBM urinary data. Generic PBPK models are well-defined compartmental models capturing toxicokinetics of xenobiotics, accounting for real-life anatomy and physiology, independently of the compound considered. The applicability of the model to the different substances is ensured by parametrising the model for each compound. The generic human PBPK model developed on INTEGRA covers major ADME processes occurring in the human body at different stages of life. The model describes in detail the absorption, distribution, metabolism, and excretion (ADME) process of both OT and its metabolite OH-o-toluidine. This data was used as an input for the toxicokinetic model of INTEGRA, properly parameterised for the assessment of internal dose of OT. Non-compound specific parameters of the model have been presented in Sarigiannis et al. [13], while further parameterisation of the compound specific parameters illustrated in Table 1 was derived on quantitative structure–activity relationship (QSAR) modelling as described in the literature [14,15]. Validation of the estimates was done by comparing the predicted OT levels with the urinary levels obtained from occupational studies reporting urinary OT concentrations, and the results have been presented in the European Human Biomonitoring Initiative (HBM4EU) deliverable “AD12.3 Exposure model testing results” [16].

Regarding the exposure reconstruction, human biomonitoring data assimilation and their conversion into intake distributions is defined formally as a computational inversion problem. In such mathematical problems, the goal is to identify the specific input distributions that best explain the observed outputs while minimising the residual error. In our case, inputs comprise spatial and temporal information on micro-environmental media concentrations of xenobiotics and ancillary information on human activities, food intake patterns, or consumer product use that results in intakes; outputs are the observed levels of biomarkers in human biospecimens. In this study, we started from the urinary levels of OT and its hydrolysed conjugate metabolites; then, exposure levels were estimated for both occupational groups and the general population.

Variability and uncertainty of the overall model were addressed through the implementation of a Bayesian Markov Chain Monte Carlo [17,18] probabilistic framework. Markov chain Monte Carlo (MCMC) techniques are numerical approximation algorithms. They are used in Bayesian inference models to sample from probability distributions through the construction of Markov chains. In Bayesian inference processes, the target distribution of each Markov chain is a marginal posterior distribution. Each Markov chain begins with a seed value; the algorithm attempting to maximise the logarithm of the non-normalised joint posterior distribution eventually arrives at each target distribution by multiple iterations. Each iteration is considered a state. A Markov chain is a random process with a finite state-space. In Markov chains, the next state depends only on the current, not past, states. The method requires defining the prior distributions, the biomonitoring data upon which it will be applied, as well as a likelihood function, defining the likelihood of the data being correct given a set of forward model parameters. The MCMC approach takes into account an acceptance criterion that considers the likelihood of the data, given parameters. The MCMC process we have used samples using algorithms based on the Metropolis Hastings (M-H) (simulated annealing) or on differential evolution algorithms. Several studies have used MCMC techniques combined with PBPK models for inverse modelling [19,20,21,22]. Key factors that introduce variability are the anthropometric parameters (i.e., bodyweight) and the metabolic constants, for which a coefficient of uncertainty equal to 30% is considered as adequate [23]. In this study, 10,000 MCMC iterations were used in the model.

### 2.3. Risk Assessment

The risk assessment was based on the SCOEL [1] OT urinary bladder cancer risk estimation using the results from a two-year rat feeding study [24]. Since no adequate epidemiological data were available, SCOEL identified data on the formation of the urinary bladder transitional-cell carcinomas in female rats as being the most relevant for hazard characterisation, and derived a benchmark dose (BMD) causing 10% tumour incidence above background level (BMD_10_) of 42.2 mg/kg bw per day. This BMD_10_ of 42.2 mg/kg bw per day was estimated to correspond to an inhaled dose of OT about 840 mg/m^3^ as an 8 h time-weighted average (TWA) in occupational exposure [1]. This assumes a body weight of 70 kg, a default inhaled volume of 10 m^3^ for an 8 h working day, and exposure of 48 weeks/year and 5 days/week. Absorption via inhalation and oral exposure was assumed the same. Allometric scaling of this dose level of 840 mg/m^3^ from rat to human using a default value of 4 [10] provided a point of departure (POD) for 10% increase in tumour risk of 210 mg/m^3^ (48 ppm). This resulted in linear OT occupational cancer risk estimations presented in Table 2 columns one and two. Using the same default numbers for a body weight and inhaled volume and mass-balance approach described in Section 2.1, we calculated the intake as mg/kg bw/d and steady-state urinary levels corresponding to the defined tumour risk levels (Table 2, columns 3 and 4).

The same BMD_10_ of 42.2 mg/kg bw per day was used as a starting point for the general population cancer risk assessment. After allometric scaling of this dose level from rat to human using a default value of 4, this provided a 10% increase in tumour risk of 10.6 mg/kg bw/d. Since the cancer data were based on the continuous oral feeding study in rats, no further dose adjustments were made. Linear OT lifetime cancer risks are presented in Table 3.

## 3. Results

### 3.1. Summary of Exposure Biomonitoring Data

The literature search resulted in four relevant papers with occupational OT exposure biomonitoring data and three relevant papers for the general population. All studies measured OT after hydrolysis of the conjugated metabolites from urinary samples. The studies concerning occupational and the general population HBM data selected in the risk assessment are presented in Table 4.

Labat et al. [25] measured urine concentrations of OT among individuals (*n* = 13) who worked in a French liquid SO_2_-plant polluted with OT. Pre-shift urine concentrations of OT had a range of 1.7 ± 1.5 μg/L, while the mean post-shift levels were 523 ± 321.6 μg/L, 95th percentile being 962 μg/L. Korinth et al. [26] assessed occupational exposure to aniline and OT among people (*n* = 51) working in the manufacturing of rubber products in Germany. The measured total urinary OT concentrations in post-shift samples were 38.6 μg/L (mean) and 292.4 μg/L (95th percentile). Li et al. [27] examined the relationship between DNA-damaging chemicals and average telomere length in 157 workers working in the Swedish rubber industry. OT levels were measured in urine samples collected during the last four hours of an eight-hour shift. Measured concentration range was 0.03–108 μg/L with a median of 0.46 μg/L. Eitaki et al. [28] studied OT-exposed workers (*n* = 36) in the Japanese pigment industry. Post-shift urinary OT measurements showed a mean concentration of 55.5 µg/L and a range of 16.5–129.12 μg/L.

Seidel [29] studied urinary excretion of aromatic amines including OT in general population in Germany. 24 h urine samples were collected from 81 non-smoking individuals aged 20–61 years. The median and maximum OT levels were found to be 61.8 and 401 ng/24 h, respectively. Another German study associated with exposure to carcinogenic aromatic amines was conducted by Riedel et al. [30]. Twenty-four urine samples were collected from 20 people (including 10 smokers). The non-smoking group had a mean OT concentration of 167 ± 199.4 ng/24 h while the concentration in the smoker group was 204.2 ± 59.1 ng/24 h. Lindner et al. [31] assessed the exposure of smokers and non-smokers to smoke constituents, including OT, by analysing 24 h urinary excretion of OT in 1631 adults (including 1223 smokers) from three countries (Germany, Switzerland, and United Kingdom). Exposure levels were lower in non-smokers than smokers, with mean OT urine concentrations of 64 ± 128 and 179 ± 497 ng/24 h respectively.

### 3.2. Reverse Calculation of External Exposure Based on Urinary Mass-Balance Approach and Generic PBPK Model

External exposures calculated for occupational and general population by using a urinary mass-balance method and a generic PBPK model for reverse dosimetry are presented in Table 5 and Table 6. In addition, corresponding cancer risks estimated using linear extrapolation as described in the Methods are presented.

### 3.3. Comparison of the Results

External dose estimations based on the urinary mass-balance method gave approximately 30% higher OT external exposure values both for workers and the general population when compared to corresponding values of the generic PBPK model. For example, in the study with the highest mean OT urinary concentration [25] the urinary mass-balance method gave a mean external exposure of 14.94 µg/kg whereas the PBPK reconstruction model gave a corresponding value of 9.96 μg/kg. The corresponding effect on OT cancer risk is in this example 50:10^6^, compared to 33:10^6^, respectively.

## 4. Discussion

A cancer risk assessment was performed for OT using existing HBM studies concerning workers and the general population. A urinary mass-balance method and a generic exposure reconstruction PBPK model were both used to estimate the external intake levels corresponding levels in urine.

In the case of OT, there are no measured correlation data on external exposure and urinary concentrations. Therefore, the urinary mass-balance method was selected, which is a rather rough method to correlate external exposure with the urinary levels. This can be further refined by using PBPK models, however, there is no specific model available for OT. A generic PBPK model was used to investigate the relation between external exposure and urinary values. The output of both models did not differ considerably; the urinary mass-balance method gave approximately 30% higher external exposure values. This difference results from the fact that the PBPK model considers exposure dynamics; hence, it can capture the intra-day variability of OT in urine and to attribute the higher concentration identified in the post-shift samples as the result of the 8 h shift. On the contrary, the biomonitored levels used for estimating intake with the urinary mass-balance method are assumed as steady-state values. If this assumption is applied on samples that have been taken from workers post-shift (representing a peak level rather than steady state level), this may result in an overestimation of external exposure. However, the 30% differences are minor when considering the uncertainties related to the cancer dose–response based on animal data.

### 4.1. Uncertainties in the Risk Assessment

The toxicokinetics of OT in humans were assumed to correspond to toxicokinetics observed in experimental animals. This is one source of uncertainty in this risk assessment. In the urinary mass-balance calculations, 75% of the OT dose (FUE) was assumed to be excreted in urine as parent compound or as hydrolysed conjugate metabolites. This was based on urinary excretion observed in s.c. dosed rats after 24 h [2]. The urinary mass-balance method used for reverse calculation assumes that the measured urinary levels represent steady-state levels. This must be considered especially in case of occupational exposure with rapidly eliminating chemicals because occupational exposure levels may vary during the working day. The half-life of OT has been reported in human plasma to be approximately four hours [32] and the available HBM data was based on post-shift urinary samples. OT is absorbed also through the human skin, which [26,33] can contribute significantly to the toxicokinetics of the compound. This should be considered when collecting/analysing HBM samples, since dermal absorption is slower when compared to inhalation exposure, resulting also in slower elimination. HBM data collected from 24 h urinary samples could better highlight the exposure. On the other hand, measurement of OT air concentrations does not consider the effect of personal protection of the worker and cannot inform us of the dermal exposure.

The carcinogenic properties of OT cause uncertainty in cancer risk assessments, since a threshold value for the cancer risk cannot be established. In this risk assessment, a linear extrapolation of cancer risk for humans was used from a BMD_10_ reference value derived for bladder tumours observed in rats. An allometric uncertainty scaling value of four was used for interspecies differences between rat and human. Linear extrapolation is considered as a conservative approach potentially resulting in the overestimation of risks at low levels. On the other hand, there is epidemiological evidence on the carcinogenicity of OT in occupationally exposed humans [3].

Individual variability in OT metabolism can have an influence on OT urinary levels. Genetic polymorphisms in human N-acetyltransferases can increase susceptibility to aromatic amine-induced cancer for slow-acetylators [34]. However, this can be considered to have a much smaller impact on the risk assessment than uncertainties in dose–response. Cigarette smoking can cause uncertainty to risk assessment since smoking elevates OT levels in the urine of smokers [31].

### 4.2. Recommendations for the Regulatory Risk Assessment

This assessment gives an example how HBM data can be used in occupational and general population risk assessments. The urinary mass-balance method may give a rough estimate of external exposure when using workers’ post-shift urinary biomonitoring data. Since OT is a rapidly metabolised chemical, the best option would be to collect consecutive urinary samples covering a whole day, i.e., 24 h (i.e., pre-shift, during shift, post-shift, evening, and next morning). The data on consecutive periods of production of urine would be also very informative for parameterising a specific PBPK model in the future. Development of a PBPK model for the chemical group of arylamines would benefit future risk assessments, especially regarding exposure reconstruction modelling.

In the future, risk assessment would benefit from well-designed biomonitoring studies. In addition, proper toxicokinetic correlation studies for controlled OT air concentration and urine excretion in humans may benefit future modelling. Moreover, exposure assessment by biomonitoring OT haemoglobin adducts can highlight exposure for longer and cumulative time periods. Mean OT Hb-adduct levels have been 10 times higher in exposed versus unexposed workers and >100 times higher than the mean levels in unexposed populations previously studied [35]. Human metabolism and kinetics of OT should be studied further.

OT has been added to the candidate list of SVHC for eventual inclusion in Annex XIV to REACH [4]. This risk assessment can be beneficial for chemical authorities as well as manufacturers in the situation that the use of OT will require authorisation in EU.

## 5. Conclusions

In conclusion, by applying the urinary mass-balance methodology and the PBPK modelling based on four OT biomonitoring studies, we found that workers exposed to OT have a cancer risk of 60 to 90:10^6^ in the worst-case scenario (i.e., the biomonitoring study with the highest urinary levels). The exposure levels and cancer risk of OT in the general population were orders of magnitude lower when compared to workers. Although there is no generally agreed acceptable cancer risk levels, usually, risk levels in the order of 1:10^5^–10^6^ for general population and around 1:10^5^ for workers are considered acceptable, but in case of workers higher levels have also been considered to be tolerable under certain circumstances [10]. The estimated external exposure levels for workers were also well below the intake levels, corresponding to the BOELV (0.5 mg/m^3^) set under EU Carcinogens and Mutagens Directive. However, results should be considered carefully because of the limited number of HBM data. There is clearly a need for further biomonitoring data on OT exposure.

## Figures and Tables

**Table 1:** Compound specific PBPK parameters for ortho-toluidine and the considered metabolite OH-o-toluidine.

	Ortho-Toluidine	OH-o-Toluidine
Tissue: Blood Partition Coefficients		
GI: Blood	3.8	0.7
Liver: Blood	1.9	0.65
Kidney: Blood	1.8	0.68
Fat: Blood	8.3	0.18
Bone: Blood	1.7	0.42
Brain: Blood	3.6	0.75
Gonads: Blood	0.79	0.83
Heart: Blood	1.6	0.57
Muscle: Blood	2.2	0.74
Skin: Blood	5.7	0.69
Lung: Blood	2.4	0.58
Fractions		
Fraction bound to plasma proteins	0.035	0.95
Fraction bound to red blood cells		0.005
Fraction of transformation from o-toluidine to OH-o-toluidine		1
Kinetic parameters		
Km (μmol/L)	27.2	
Vmax (μmol/h)	2835.3	
Clearances		
Kidney clearance rate (L/min)	0	0.17
Absorption GI tract		
Absorption fraction from GI tract	1	
Absorption rate in GI tract	1	

**Table 2:** Ortho-toluidine linear cancer risk estimations, corresponding to occupational exposure to specific air levels, calculated daily occupational intake, and calculated urinary steady-state levels.

Tumour Risk	Ortho-Toluidine Concentration, mg/m^3^ (ppm)	Occupational Intake (mg/kg bw/d) ^1^	Steady-State Urinary Level (mg/L) ^2^
1:10	210 (48)	30	1000
1:1000	2.1 (0.48)	0.3	10
1:10,000	0.21 (0.048)	0.03	1
1:100,000	0.021 (0.0048)	0.003	0.1
1:1,000,000	0.0021 (0.00048)	0.0003	0.01

^1^ Converted to mg/bw/working day (as occupational exposure 8 h/day, 5 d/week, 70 kg worker, inhaling 10 m^3^ of air during the working day). ^2^ Calculated urinary steady-state levels with mass-balance equation (75% of parent compound excreted to the urine including hydrolysed conjugate metabolites (FUE), 1.5 L/day of 24 h urinary volume (V24) and 70 kg body weight (BW).

**Table 3:** Ortho-toluidine linear cancer risk estimations for general population, corresponding to specific lifetime cancer risk, and calculated urinary steady-state levels.

Tumour Risk	Ortho-Toluidine Intake (mg/kg bw/d)	Steady-State Urinary Level (mg/L) ^1^
1:10	10.6	371
1:1000	0.106	3.71
1:10,000	0.0106	0.371
1:100,000	0.00106	0.0371
1:1,000,000	0.000106	0.00371

^1^ Calculated urinary steady-state levels with mass-balance equation (75% of parent compound excreted to the urine including hydrolysed conjugate metabolites (FUE), 1.5 L/day of 24 h urinary volume (V24) and 70 kg body weight (BW).

**Table 4:** Human biomonitoring studies for ortho-toluidine in occupational settings and background concentrations of the general populations.

Study Origin	Urine Sample Type (*n*)	Urine Ortho-Toluidine (Range)	Reference
Workers, Liquid SO_2_ plant, France	Post-shift (13)	Mean: 523 µg/L, P95: 962 µg/L (<LOD-984.1 µg/L)	[25]
Workers, Rubber industry, Germany	Post-shift (51)	Mean: 38.6 µg/L, P95: 292.4 µg/L (<LOD-292.4 µg/L)	[26]
Workers, Rubber industry, Sweden	Post-shift (157)	Median: 0.46 µg/L, (0.03–108 µg/L)	[27]
Workers, Pigment industry, Japan	Post-shift (36)	Mean: 55.5 µg/L, (<LOD-129.12 µg/L)	[28]
General population, Germany	24 h urine (81)	Median: 61.8 ng/24 h, Max: 401 ng/24 h	[29]
General population, Germany	24 h urine (20)	Mean (non-smokers): 167 ± 199.4 ng/24 h	[30]
		Mean (smokers): 204.2 ± 59.1 ng/24 h.	
General population, Germany, Switzerland, United Kingdom	24 h urine (1631)	Mean (non-smokers): 64 ± 128 ng/24 h	[31]
		Mean (smokers): 179 ± 497 ng/24 h	

LOD = limit of detection, *n* = number of study participants, P95 = 95th percentile.

**Table 5:** Estimated occupational cancer risk values for o-toluidine using a urinary mass-balance method and a PBPK model in INTEGRA.

	Urinary Ortho-Toluidine (μg/L)	External Ortho-Toluidine Exposure (μg/kg bw/d)	Estimated Cancer Risk
Urinary mass-balance			
Labat et al. [25]	Mean: 523, P95: 962	Mean: 14.94, P95: 27.49	50–92:10^6^
Korinth et al. [26]	Mean: 38.6, P95: 292.4	Mean: 1.10, P95: 8.35	4–28:10^6^
Li et al. [27]	Median: 0.46, Max: 108	Median: 0.013, Max: 3.09	0.04–10:10^6^
Eitaki et al. [28]	Mean: 55.5, Max: 129.1	Mean: 1.6, Max: 3.69	5–12:10^6^
PBPK model			
Labat et al. [25]	Mean: 523, P95: 962	Mean: 9.96, P95: 18.74	33–62:10^6^
Korinth et al. [26]	Mean: 38.6, P95: 292.4	Mean: 0.70, P95: 5.57	2–19:10^6^
Li et al. [27]	Median: 0.46, Max: 108	Median: 0.012, Max: 2.06	0.04–7:10^6^
Eitaki et al. [28]	Mean: 55.5, Max: 129.1	Mean: 1.1, Max: 2.5	4–8:10^6^

P95 = 95th percentile.

**Table 6:** Estimated lifetime cancer risk values of o-toluidine for the general population using a urinary mass-balance method and a PBPK model in INTEGRA.

	Urinary Ortho-Toluidine (ng/24 h)	External Ortho-Toluidine Exposure (μg/kg bw/d)	Estimated Cancer Risk
Urinary mass-balance			
Seidel [29]	non-smokers: Mean: 61.8, Max: 401	non-smokers: Mean: 0.0012, Max: 0.008	0.0011–0.0072:10^6^
Riedel et al. [30]	non-smokers: 167 ± 199.4	non-smokers: 0.0032 ± 0.004	0.001–0.01:10^6^
	smokers: 204.2 ± 59.1	smokers: 0.0039 ± 0.001	0.004–0.007:10^6^
Lindner et al. [31]	non-smokers: 64 ± 128	non-smokers: 0.0012 ± 0.0024	0.002–0.005:10^6^
	smokers: 179 ± 497	smokers: 0.0034 ± 0.0095	0.009–0.018:10^6^
PBPK model			
Seidel [29]	non-smokers: Mean: 61.8, Max: 401	non-smokers: Mean: 0.00078, range 0.00012–0.00514	0.0001–0.005:10^6^
Riedel et al. [30]	non-smokers: 167 ± 199.4	non-smokers: Mean 0.00133, range 0.0001–0.0031	0.0001–0.003:10^6^
Lindner et al. [31]	non-smokers: 64 ± 128	non-smokers: Mean 0.0008, range 0.00006–0.0019	0.0001–0.0018:10^6^

## Data Availability

The data presented in this study are available publicly.

## References

[1] (2017). Recommendation from the Scientific Committee on Occupational Exposure Limits. o-Toluidine, 2-methylaniline. SCOEL/REC/301. https://op.europa.eu/s/v5bh.

[2] (2004). SIDS Initial Assessment Report (SIAR) on O-Toluidine.

[3] (2012). A review of human carcinogens: Chemical agents and related occupations. IARC Monogr. Eval. Carcinog. Risks Hum..

[4] ECHA—Candidate List of substances of Very High Concern for Authorisation. https://echa.europa.eu/candidate-list-table.

[5] (2014). Report on Carcinogens, Monograph on Ortho-Toluidine; National Toxicology Program.

[6] European Commission, Directive (EU) 2019/130 of the European Parliament and of the Council of 16 January 2019 Amending Directive 2004/37/EC on the Protection of Workers from the Risks Related to Exposure to Carcinogens or Mutagens at Work. https://eur-lex.europa.eu/legal-content/EN/TXT/?uri=uriserv:OJ.L_.2019.030.01.0112.01.ENG.

[7] Kütting B., Göen T., Schwegler U., Fromme H., Uter W., Angerer J., Drexler H. (2009). Monoarylamines in the general population—A cross-sectional population-based study including 1004 Bavarian subjects. Int. J. Hyg. Environ. Health.

[8] Weiss T., Angerer J. (2005). Belastung der Bevölkerung der Bundesrepublik Deutschland durch nitroaromatischen Verbindungen—Der Einfluss von Ernährung und Bekleidung.

[9] (2012). MAK- und BAT-Werte-Liste. Grenzwerte in Biologischem Material zu o-Toluidin. Addendum zu o-Toluidin.

[10] (2012). ECHA—Guidance on Information Requirements and Chemical Safety Assessment Chapter R.8: Characterisation of Dose [Concentration]-Response for Human Health. ECHA-2010-G-19-EN. https://echa.europa.eu/documents/10162/13632/information_requirements_r8_en.pdf/e153243a-03f0-44c5-8808-88af66223258.

[11] Angerer J., Aylward L.L., Hays S.M., Heinzow B., Wilhelm M. (2011). Human biomonitoring assessment values: Approaches and data requirements. Int. J. Hyg. Environ. Health.

[12] Sarigiannis D., Karakitsios S., Gotti A., Loizou G., Cherrie J., Smolders R., De Brouwere K., Galea K., Jones K., Handakas E. Integra: From global scale contamination to tissue dose. Proceedings of the 7th International Congress on Environmental Modelling and Software: Bold Visions for Environmental Modeling, iEMSs.

[13] Sarigiannis D., Karakitsios S.P., Handakas E., Simou K., Solomou E., Gotti A. (2016). INTEGRAted exposure and risk characterization of bisphenol-A in Europe. Food Chem. Toxicol..

[14] Papadaki K.C., Karakitsios S.P., Sarigiannis D.A. (2017). Modeling of adipose/blood partition coefficient for environmental chemicals. Food Chem. Toxicol..

[15] Sarigiannis D.A., Papadaki K., Kontoroupis P., Karakitsios S.P. (2017). Development of QSARs for parameterizing Physiology Based ToxicoKinetic models. Food Chem. Toxicol..

[16] Sarigiannis D.A. (2019). AD12.3 Exposure Model Testing Results, HBM4EU Project. https://www.hbm4eu.eu/work-packages/additional-deliverable-12-3-exposure-model-testing-results/.

[17] Gelman A., Rubin D.B. (1996). Markov chain Monte Carlo methods in biostatistics. Stat. Methods Med. Res..

[18] Gilks W., Spiegelhalter D., Richardson S. (1996). Markov Chain Monte Carlo in Practice.

[19] Chen C.C., Shih M.C., Wu K.Y. (2010). Exposure estimation using repeated blood concentration measurements. Stoch. Environ. Res. Risk Assess..

[20] Georgopoulos P.G., Sasso A.F., Isukapalli S.S., Lioy P.J., Vallero D.A., Okino M., Reiter L. (2009). Reconstructing population exposures to environmental chemicals from biomarkers: Challenges and opportunities. J. Expo. Sci. Environ. Epidemiol..

[21] Lyons M.A., Yang R.S., Mayeno A.N., Reisfeld B. (2008). Computational toxicology of chloroform: Reverse dosimetry using Bayesian inference, Markov chain Monte Carlo simulation, and human biomonitoring data. Environ. Health Perspect..

[22] McNally K., Cotton R., Cocker J., Jones K., Bartels M., Rick D., Price P., Loizou G. (2012). Reconstruction of exposure to m-xylene from human biomonitoring data using PBPK modelling, bayesian inference, and Markov chain Monte Carlo simulation. J. Toxicol..

[23] Clewell R., Clewell H. (2008). Development and specification of physiologically based pharmacokinetic models for use in risk assessment. Regul. Toxicol. Pharmacol..

[24] (1979). Bioassay of o-Toluidine Hydrochloride for Possible Carcinogenicity (CAS No. 636-21-5).

[25] Labat L., Thomas J., Dehon B., Humbert L., Leleu B., Nisse C., Lhermitte M. (2006). Evalution d’une exposition professionnelle à l’ortho-toluidine par chromatographie phase gazeuse couplée à la spectrométrie de masse. Acta Clin. Belg..

[26] Korinth G., Weiss T., Penkert S., Schaller K.H., Angerer J., Drexler H. (2007). Percutaneous absorption of aromatic amines in rubber industry workers: Impact of impaired skin and skin barrier creams. Occup. Environ. Med..

[27] Li H., Jönsson B.A.G., Lindh C.H., Albin M., Broberg K. (2011). N-nitrosamines are associated with shorter telomere length. Scand. J. Work Environ. Health.

[28] Eitaki Y., Nakano M., Kawai T., Omae K., Takebayashi T. (2019). Biological monitoring of o-toluidine in urine pretreated by an enzymatic deconjugation method. J. Occup. Health.

[29] Seidel A. (2005). Ermittlung von Quellen für das Vorkommen von Nitro-/Aminoaromaten im Urin von Nichtrauchern.

[30] Riedel K., Scherer G., Engl J., Hagedorn H.W., Tricker A.R. (2006). Determination of three carcinogenic aromatic amines in urine of smokers and nonsmokers. J. Anal. Toxicol..

[31] Lindner D., Smith S., Leroy C.M., Tricker A.R. (2011). Comparison of exposure to selected cigarette smoke constituents in adult smokers and nonsmokers in a European, multicenter, observational study. Cancer Epidemiol. Biomark. Prev..

[32] Richter E. (2015). Biomonitoring of human exposure to arylamines. Front. Biosci..

[33] Lüersen L., Wellner T., Koch H.M., Angerer J., Drexler H., Korinth G. (2006). Penetration of b-naphthylamine and o-toluidine through human skin in vitro. Arch Toxicol..

[34] Golka K., Prior V., Blaszkewicz M., Bolt H.M. (2002). The enhanced bladder cancer susceptibility of NAT2 slow acetylators towards aromatic amines: A review considering ethnic differences. Toxicol. Lett..

[35] Hanley K.W., Viet S.M., Hein M.J., Carreón T., Ruder A.M. (2012). Exposure to o-toluidine, aniline, and nitrobenzene in a rubber chemical manufacturing plant: A retrospective exposure assessment update. J. Occup. Environ. Hyg..

